# Serum Secretogranin III Concentrations Were Increased in Subjects with Metabolic Syndrome and Independently Associated with Fasting Plasma Glucose Levels

**DOI:** 10.3390/jcm8091436

**Published:** 2019-09-11

**Authors:** Chun-Chung Lin, Kai-Pi Cheng, Hao-Chang Hung, Chung-Hao Li, Ching-Han Lin, Chih-Jen Chang, Che-Yuan Hu, Hung-Tsung Wu, Horng-Yih Ou

**Affiliations:** 1 Division of Endocrinology and Metabolism, Department of Internal Medicine, National Cheng Kung University Hospital, College of Medicine, National Cheng Kung University, Tainan 70101, Taiwan; crystal_qoo@hotmail.com (C.-C.L.); supercabyhome@yahoo.com.tw (K.-P.C.); haochang.hung@gmail.com (H.-C.H.); cyclops0113@yahoo.com.tw (C.-H.L.); 2 Department of Family Medicine, National Cheng Kung University Hospital, College of Medicine, National Cheng Kung University, Tainan 70101, Taiwan; smallhear@gmail.com (C.-H.L.); changcj.ncku@gmail.com (C.-J.C.); 3 Department of Health Management Center, National Cheng Kung University Hospital, National Cheng Kung University, Taiwan 70101, Taiwan; 4 Department of Urology, National Cheng Kung University Hospital, College of Medicine, National Cheng Kung University, Tainan 70101, Taiwan; greatoldhu@gmail.com; 5 Graduate Institute of Metabolism and Obesity Sciences, College of Nutrition, Taipei Medical University, Taipei 11031, Taiwan

**Keywords:** free fatty acid, hyperglycemia, metabolic syndrome, obesity, secretogranin III

## Abstract

Secretogranin III (SCG3) plays a crucial role in the biogenesis of secretory granules in endocrine cells, and thus affects glucose homeostasis by regulating insulin secretion by pancreatic beta cells. Insulin resistance and compensatory hyperinsulinemia are hallmarks of metabolic syndrome (MetS). However, the role of SCG3 in MetS remains unclear. Therefore, we investigated the relationship between serum SCG3 levels and metabolic parameters in subjects with and without MetS. This was a case control study, and 295 subjects were recruited. Serum SCG3 concentrations were compared between groups. Associations between SCG3 levels and clinico-metabolic parameters were also examined. We found serum SCG3 levels were higher in the MetS group than non-MetS group (122.6 ± 79.2 vs. 90.6 ± 58.5 nmol/L, *p* = 0.009). Specifically, elevated SCG3 levels were found in subjects with high fasting plasma glucose (FPG) levels, central obesity, or hypertriglyceridemia. Additionally, MetS was an independent factor of serum SCG3 levels in multivariate linear regression analyses. Moreover, FPG, free fatty acids, and waist circumference were positively associated with serum SCG3 concentrations after adjusting for insulin levels, high-sensitivity C-reactive protein, and cardiovascular risk factors. In conclusion, serum SCG3 concentrations were higher in subjects with MetS and were independently associated with FPG levels.

## 1. Introduction

Metabolic syndrome (MetS) is a cluster of interrelated risk factors which include an elevated waist circumference (WC), hypertriglyceridemia, reduced high-density lipoprotein cholesterol (HDL-C), high blood pressure, and dysglycemia [1]. Given one quarter of the global population affected [2], and the fact that subjects with MetS are at increased risk of diabetes, cardiovascular events, and death [3,4], MetS has become an important public health issue worldwide. Notably, insulin resistance (IR) and subsequent compensatory hyperinsulinemia were proven to be the core pathophysiology of MetS and related metabolic disorders [5,6], and thus can be the target of therapy.

Secretogranin III (SCG3) is a member of the granin family of proteins, which consists of chromogranins, secretogranins, and other related proteins [7]. The granin family exists in a variety of endocrine and neuroendocrine cells [8,9], and previous studies identified SCG3 in adrenal neuroendocrine cell lines [10], the hypothalamus [11,12], pituitary cell lines, pancreatic beta cell lines [13], and insulin-secreting cells of pancreatic tissues in rats [14] and humans [15]. Regarding its physiologic function, SCG3, which interacts with chromogranin A (CgA), is considered to play a crucial role in regulating biogenesis of secretory granules that contain bioactive peptides, hormones, growth factors, and neurotransmitters in endocrine cells [7,16]. Acting as a bridge of peptides and a trans-Golgi network, it sorts these prohormones into secretory granules [16,17]. As for insulin secretion, SCG3 also had some roles in assisting insulin processing, thus affecting glucose homeostasis [18,19]. It has been shown that significantly increased SCG3 levels were detected in serum-free media conditioned with non-responsive high-passage MIN-6 cells, a model of dysfunctional beta cells. These data implied that secretory granules can no longer form properly, thus decreasing the insulin-secreting ability of beta cells, when SCG3 was massively released from, but did not remain inside, those cells [18]. Additionally, proinsulin was found to be insufficiently converted into the mature form, insulin, in islet cells, thus leading to hyperglycemia, in SCG3-knockout mice fed a high-fat/high-sucrose diet [19]. This finding suggests the role of SCG3 in proteolytic conversion of the inactive prohormone to the active hormone [19]. Taking those results together, SCG3 may be involved in glucose homeostasis by regulating insulin processing and secretions by pancreatic beta cells. Since IR and subsequent insulin hypersecretion are very much a part of MetS, we assumed a certain role of SCG3 in subjects with MetS. However, no study has adequately explored this issue. Therefore, we conducted a case control study to investigate the relationship between serum SCG3 levels and metabolic parameters in subjects with and those without MetS.

## 2. Materials and Methods

### 2.1. Study Subjects

The present study was a case-control study. All protocols of the study were approved by the Human Experiment and Ethics Committee of National Cheng Kung University Hospital (NCKUH B-ER-102-418), and written informed consent was obtained from all participants. All subjects aged 20~80 years who were admitted to the Preventive Health Center of National Cheng Kung University Hospital for a health checkup from June 2007 to July 2009 were screened. Individuals with the following conditions or diseases were excluded: (1) Type 1 diabetes mellitus; (2) receiving insulin therapy, glucagon like peptide-1, or oral antidiabetic drugs; (3) taking drugs that affect glucose homeostasis, including corticosteroids, thiazides, atypical antipsychotic drugs, or sympathomimetic agents; (4) being pregnant; (5) having an acute infection, such as pneumonia, urinary tract infection, soft-tissue infection, or sepsis; (6) having experienced a recent event of acute coronary syndrome, cerebrovascular accident, or pancreatitis during the past 3 months; (7) having any advanced malignant diseases or other major diseases with generalized inflammation contraindicating this study; (8) taking lipid-lowering medications; and (9) taking antihypertensive drugs.

### 2.2. Data Collection

With the help of a trained assistant, body height (to the nearest 0.1 cm) and weight (to the nearest 0.1 kg) measurements of each subject were conducted. The body-mass index (BMI) (in kg/m^2^) was calculated as weight (in kilograms) divided by body height (in meters) squared. The WC was measured on bare skin midway between the lower rib margin and the iliac crest at the end of normal expiration. Additionally, after a 15-minute rest, the systolic blood pressure (SBP) and diastolic blood pressure (DBP) of participants were checked twice with an interval of at least 5 minutes in a supine position in a quiet environment.

After an overnight fast for at least 12 hours, all subjects received blood tests including fasting plasma glucose (FPG), glycated hemoglobin A1c (HbA1C), renal function (creatinine), liver enzymes (aspartate aminotransferase (AST) and alanine aminotransferase (ALT)), and lipid profiles (including total cholesterol (TC), triglycerides (TGs), and high- (HDL-C) and low-density lipoprotein cholesterol (LDL-C)). In addition, using commercial enzyme-linked immunosorbent assay (ELISA) kits, serum concentrations of SCG3 (Mybiosource, San Diego, CA, USA; with an intra-assay coefficient of variation (CV) of 8% and interassay CV of 10%), insulin (Mercodia AB, Uppsala, Sweden; with an intra-assay CV of 4% and an interassay CV of 2.6%), high-sensitivity C-reactive protein (hsCRP) (Immunology Consultants Laboratory, Newberg, OR; with an intra-assay CV of 2.9% and an interassay CV of 4.7%), and free fatty acid (FFA) levels (BioVision, San Francisco, CA, USA; with an intra-assay CV of 5.9% and an interassay CV of 5.6%) were determined. Moreover, after the fasting blood test was obtained, a subsequent 75-g oral glucose tolerance test (OGTT), which collected data on the plasma glucose level at 120 minutes, was carried out.

To calculate the estimated glomerular filtration rate (eGFR, mL/min/1.73 m^2^), the modification of diet in renal disease (MDRD) equation was used. Furthermore, the homeostasis model assessment of IR (HOMA-IR) was applied to investigate IR with the formula HOMA-IR = fasting insulin in mU/L × FPG in mmol/L/405 [20].

### 2.3. Definition of MetS

MetS is diagnosed if three or more of the following five criteria are met [1,21]: (1) A WC of ≥90 cm in men and ≥80 cm in women; (2) SBP of ≥130 mmHg or DBP of ≥85 mmHg; (3) TGs of ≥1.7 mmol/L; (4) HDL-C of <1.03 mmol/L in men and <1.29 mmol/L in women; and (5) FPG of ≥5.6 mmol/L. Subjects who fulfilled the above definition were classified as the MetS group; all other subjects were designated the non-MetS group.

### 2.4. Statistical Analysis

The Windows version of the Statistical Package for the Social Sciences (SPSS version. 21.0; SPSS, Chicago, IL, USA) was used for all statistical analyses. All normally distributed continuous variables are expressed as the mean ± standard deviation (SD), and categorical variables as percentages. All participants were divided into a MetS group and non-MetS group. Chi-square tests and Student’s *t*-tests were respectively used to analyze differences in categorical and continuous variables between the two groups. In addition, Pearson’s correlation coefficients were analyzed to assess the strength of associations between serum SCG3 levels and clinical parameters of all participants. Furthermore, we conducted multiple linear regression analyses to identify independent factors related to SCG3 concentrations, using stepwise and backward variable selection strategies. A *p* value of <0.05 was considered statistically significant.

## 3. Results

In total, 295 subjects, including 129 women and 166 men, were enrolled in the study. Among them, 18.3% of participants (*n* = 54) were grouped into the MetS group, and 81.7% of participants (*n* = 241) belonged to the non-MetS group. In addition, 90, 165, and 40 subjects were diagnosed with normal glucose tolerance, prediabetes (impaired fasting glucose or impaired glucose tolerance), and diabetes, respectively, according to the results of FPG and the OGTT.

**Figure 1:**
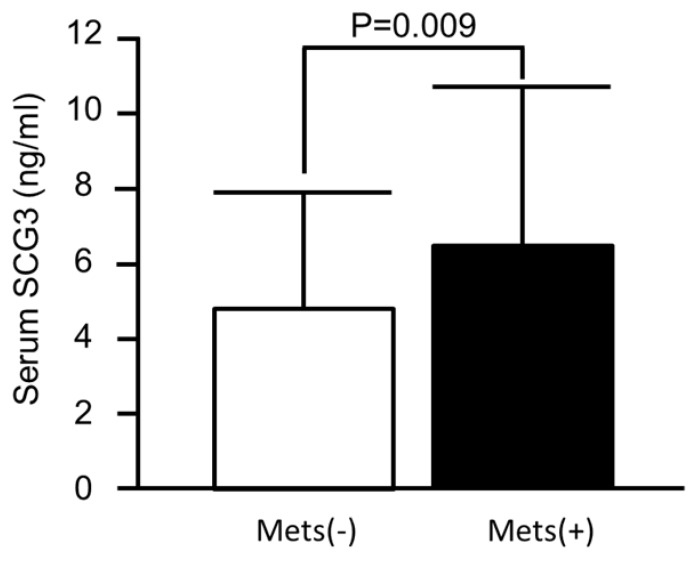
Comparison of serum secretogranin III levels between subjects without and those with metabolic syndrome (MetS).

**Figure 2:**
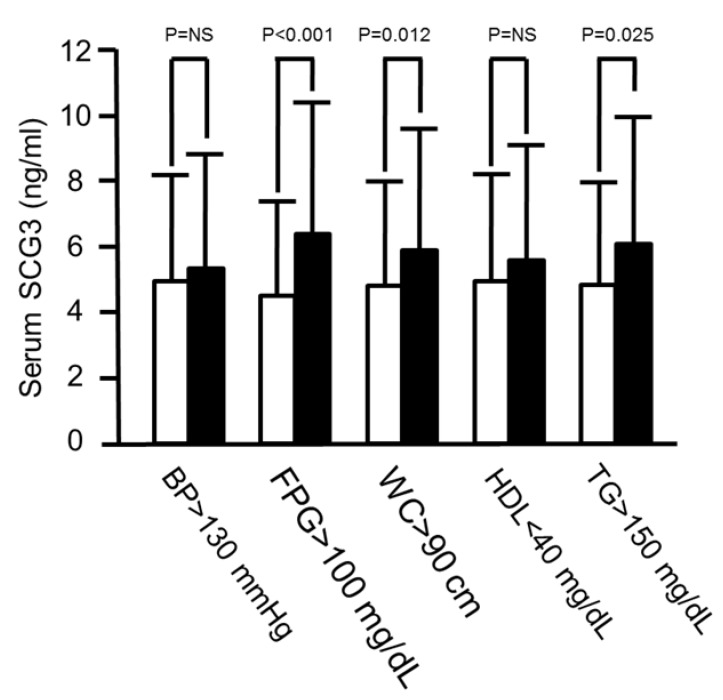
Comparisons of serum secretogranin III levels between subjects with/without each component of metabolic syndrome.

Clinical characteristics of the MetS group and non-MetS group were compared, as shown in Table 1. There were significant differences in WC (87.9 ± 8.6 vs. 79.6 ± 8.3 cm, *p* < 0.001), BMI (25.3 ± 3.1 vs. 22.5 ± 2.7 kg/m^2^, *p* < 0.001), SBP (138.2 ± 15.1 vs. 125.1 ± 18.2 mmHg, *p* < 0.001), DBP (79.8 ± 8.9 vs. 72.3 ± 10.3 mmHg, *p* < 0.001), FPG (7.9 ± 3.7 vs. 5.2 ± 1.6 mmol/L, *p* < 0.001), HDL-C (1.2 ± 0.4 vs. 1.5 ± 0.4 mmol/L, *p* < 0.001), TGs (2.2 ± 1.3 vs. 1.1 ± 0.5 mmol/L, *p* < 0.001), insulin (3.6 ± 2.5 vs. 2.4 ± 3.5 mU/L, *p* < 0.001), HOMA-IR (1.2 ± 0.9 vs. 0.5 ± 0.5, *p* < 0.001), glucose level at two hours during the OGTT (12.1 ± 5.6 vs. 8.6 ± 3.9 mmol/L, *p* < 0.001), hsCRP (52.4 ± 65.7 vs. 29.5 ± 61.9 nmol/L, *p* < 0.001), and FFAs (5.6 ± 5.0 vs. 3.8 ± 3.5 µM, *p* < 0.001) between the MetS group and non-MetS group. Moreover, there were higher serum SCG3 levels in the MetS group than in the non-MetS group (122.6 ± 79.2 vs. 90.6 ± 58.5 nmol/L, *p*=0.009) (Figure 1). Of note, when we assessed SCG3 levels in patients with and those without each respective single component of MetS, higher serum SCG3 levels were only found in subjects with high FPG (120.7 ± 75.5 vs. 84.9 ± 54.7 nmol/L, *p* < 0.001), elevated WC (111.3 ± 69.8 vs. 90.6 ± 60.4 nmol/L, *p* = 0.012), or hypertriglyceridemia (115.1 ± 73.6 vs. 92.4 ± 60.4 nmol/L, *p* = 0.025), but not elevated blood pressure (100.0 ± 66.0 vs. 94.3 ± 62.3 nmol/L, *p* = 0.53) or reduced HDL-C levels (105.6 ± 66.0 vs. 94.3 ± 62.3 nmol/L, *p* = 0.47) (Figure 2).

Correlations between serum SCG3 levels and clinical parameters of all participants are shown in Table 2. The SCG3 concentration was positively related to the WC, BMI, FPG, glycated hemoglobin, logTGs, and glucose level at two hours during the OGTT. To further determine independent factors associated with serum SCG3 levels, multivariate linear regression analyses were performed. Table 3 summarizes findings of both the stepwise and backward selection strategies. The presence of MetS was an independent factor of the serum SCG3 level (β = 0.190, 95% confidence interval (CI) = 0.665~2.644, *p* = 0.001) after adjusting for age and sex (model 1). In addition, this independent association was still found (β = 0.164, 95% CI = 0.410~2.461, *p* = 0.006) after further adjusting for FFAs, hsCRP, insulin, the eGFR, and GPT (model 2). It is particularly noteworthy that serum FFA levels were also an independent factor associated with serum SCG3 levels in model 2 (β = 0.179, 95% CI = 0.052~0.260, *p* = 0.003). Moreover, when we further took every single component of MetS into consideration, FPG (β = 0.216, 95% CI = 0.683~2.490, *p* = 0.001) and WC (β = 0.133, 95% CI = 0.114~1.897, *p* = 0.027), but not TGs, HDL-C, or SBP, were proven to be independently associated with serum SCG3 levels (model 3).

## 4. Discussion

To the extent of our knowledge, this is the first report providing evidence that serum SCG3 levels are higher in subjects with MetS. Specifically, there were higher serum SCG3 levels in subjects with high FPG levels, larger WCs, or hypertriglyceridemia. Additionally, the presence of MetS, FPG, FFAs, and WC were independently associated with serum SCG3 levels after adjusting for the insulin level, hsCRP, and other cardiovascular risk factors.

Secretory granules are organelles that contain bioactive peptide hormones in endocrine cells. Their formation and further secretion from cells involve sophisticated processes, in which SCG3 is indispensable. First, SCG3 binds to prohormone-chromogranin A aggregates. Then SCG3 acts as a bridge of the core hormone aggregates and the cholesterol component of the trans-Golgi network, thus sorting the peptide prohormones into secretory granules [16,17]. In parallel, some processing enzymes, such as carboxypeptidase E and prohormone-converting enzymes, gather in this cholesterol-rich membrane platform. Then the prohormones are converted to their mature forms. Notably, the SCG3-CPE interaction helps this prohormone processing [17]. In line with this concept, previous studies indicated that SCG3 had some roles in assisting the formation of insulin-containing secretory granules and insulin processing, thus affecting glucose homeostasis [18,19]. In our study, serum SCG3 concentrations were positively related to FPG, glycated hemoglobin, and glucose levels at two hours of the OGTT. Additionally, FPG remained an independent factor associated with serum SCG3 levels even after adjusting for other metabolic factors. Therefore, our results indicate that there was a compensatory increase in SCG3 formation which enhanced insulin secretion to overcome the hyperglycemia produced by IR. However, serum SCG3 concentrations were only positively correlated with the glycemic level, but not the insulin level, in our study. It was shown that it was the compensatory increase in insulin secretion that maintained normal glucose tolerance in a condition of IR. On the contrary, failure of compensatory hypersecretion, or even a decrease in insulin secretion, led to a status of impaired glucose tolerance or even overt diabetes [22]. In the present study, around 70% of participants were prediabetic or overtly diabetic. Thus, we speculated that while increased SCG3 synthesis facilitated the formation of secretory granules, there were still other factors hindering the successful compensation for insulin hypersecretion, which resulted in dysglycemia. After all, mechanisms of the formation and further secretion of insulin-containing secretory granules are complicated, and SCG3 is just a part of it. Similarly, our results showed that FFAs were independently associated with SCG3 concentrations. Since FFAs were shown to increase insulin secretion by beta cells to compensate for IR [23,24]. Nolan et al. showed in their study that exogenous palmitate markedly potentiated glucose-stimulated insulin secretion (GSIS) in ZF islets, allowing robust secretion at physiological glucose levels [23]. It has also been reported that FFA signaling via FFAR1 involves increases in intracellular Ca2+ for insulin secretion [24,25]. We here postulate that elevated FFAs increase SCG3 secretion because FFAs were shown to increase insulin secretion by beta cells to compensate for insulin resistance. However, future studies are needed to examine our postulate.

Interestingly, several loci on chromosomes were linked with MetS, diabetes, and obesity, including regions on chromosome 15q [26,27].The gene encoding SCG3, which is located on chromosome 15q as well, was therefore associated with obesity. However, there were inconsistent findings regarding the role of SCG3 in weight change and appetite regulation in previous studies. Tanabe et al. indicated that SCG3 was expressed along with several kinds of appetite-stimulating peptides, such as neuropeptide Y (NPY), orexin, and melanin-concentrating hormone (MCH), in the hypothalamus [12]. Decreases in appetite-regulating hormones, which resulted in less food intake and subcutaneous fat amounts, were found in subjects with increased SCG3 expression led by a single-nucleotide polymorphism [12]. In other words, decreased SCG3 levels may be associated with a higher risk of obesity. On the contrary, another study by Hotta et al. found that not only the amounts of orexin, NPYs and MCH, but also the SCG3 level in the hypothalamus, increased after a 24-hour fast. The authors therefore considered the increased expressions of SCG3 and other orexigenic hormones as a compensatory event to acute weight changes and energy deficits. Nevertheless, in the same study, there was no significant change in SCG3 levels when subjects experienced chronic weight gain induced by a high-fat diet [11]. In our study, WC and the BMI were positively associated with serum SCG3 levels. In addition, WC remained a factor positively associated with serum SCG3 levels in the multivariate regression analysis. In our opinion, there are two possible hypotheses explaining the current findings. First, in line with the proposition from Tanabe et al. [12], increased SCG3 levels in subjects with an elevated WC might be a compensatory strategy to reduce orexigenic hormones and subcutaneous fat accumulation. Alternatively, the increased expression of SCG3 might thus facilitate the formation of secretory granules, which contain a number of appetite-stimulating hormones, which make subjects gain weight. Due to the design of our study, further efforts are still needed to discover the detailed mechanisms in this field.

There are some limitations in this work. First, as previous studies suggested that SCG3 may derive from hypothalamus-pituitary-adrenal axis, dysregulation in this axis might affect circulating SCG3 levels. Regarding this, we did not measure and adjust plasma cortisol levels in the present study to exclude this possibility. However, we excluded subjects with conditions of acute or chronic inflammation/infection in our study, and also adjusted hsCRP in the regression analysis. The resulting independent association between MetS and SCG3 levels implies the HPA axis had little effect. Second, to minimize the effects of external factors, the recruited population did not take oral or injectable antidiabetic drugs, lipid-lowering medications, or antihypertensive agents. Therefore, our findings have not been verified in subjects with MetS who were taking such medications. Third, all study subjects were Taiwanese, and thus our results might not be able to be applied to other ethnicities.

## 5. Conclusions

Serum SCG3 levels were higher in subjects with MetS. In addition, FPG, FFAs, and WC were important factors independently associated with serum SCG3 concentrations. These findings support the possibility of SCG3 being a promising target for controlling dysglycemia. However, further work is still needed to investigate the exact mechanisms involved in the elevated serum SCG3 levels and their clinical implications in MetS.

## Figures and Tables

**Table 1:** Comparisons of clinical characteristics between groups.

Variables	Non-Metabolic Syndrome	Metabolic Syndrome	*p*
*n*	241	54	
Age (years)	61.9 ± 12.2	62.4 ± 10.8	0.77
Female (%)	41.1	50	0.29
WC (cm)	79.6 ± 8.3	87.9 ± 8.6	<0.001
BMI (kg/m^2^)	22.5 ± 2.7	25.3 ± 3.1	<0.001
eGFR (mL/min/1.73m^2^)	92.7 ± 20.3	89.2 ± 17.4	0.25
creatinine (µmol/L)	79.6 ± 17.7	79.6 ± 17.7	0.93
SBP (mmHg)	125.1 ± 18.2	138.2 ± 15.1	<0.001
DBP (mmHg)	72.3 ± 10.3	79.8 ± 8.9	<0.001
FPG (mmol/L)	5.2 ± 1.6	7.9 ± 3.7	<0.001
GLU_120 (mmol/L)	8.6 ± 3.9	12.1 ± 5.6	<0.001
HbA1c	6.0 ± 1.0	7.2 ± 2.1	<0.001
AST (U/L)	25.2 ± 8.5	27.9 ± 13.2	0.14
ALT (U/L)	23.0 ± 12.7	28.5 ± 17.0	0.03
HDL-C (mmol/L)	1.5 ± 0.4	1.2 ± 0.4	<0.001
LDL-C (mmol/L)	3.2 ± 0.9	3.4 ± 1.1	0.17
Triglyceride (mmol/L)	1.1 ± 0.5	2.2 ± 1.3	<0.001
HOMA-IR	0.5 ± 0.5	1.2 ± 0.9	<0.001
Insulin (mU/L)	2.4 ± 3.5	3.6 ± 2.5	<0.001
hsCRP (nmol/L)	29.5 ± 61.9	52.4 ± 65.7	<0.001
FFAs (µM)	3.8 ± 3.5	5.6 ± 5.0	<0.001

WC, waist circumference; BMI, body-mass index; eGFR, estimated glomerular filtration rate; SBP, systolic blood pressure; DBP, diastolic blood pressure; FPG, fasting plasma glucose; GLU_120, glucose level at two hours during an oral glucose tolerance test; HbA1c, glycated hemoglobin A1c; AST, aspartate aminotransferase; ALT, alanine aminotransferase; HDL-C, high-density lipoprotein cholesterol; LDL-C, low-density lipoprotein cholesterol; HOMA-IR, homeostasis model assessment of insulin resistance; hsCRP, high-sensitivity C-reactive protein; FFAs, free fatty acids.

**Table 2:** Correlations of serum secretogranin III (SCG3) levels and clinical parameters in all subjects.

Variables	*r*	*p* Value
Age (years)	−0.087	0.09
FPG (mmol/L)	0.149	0.003
GLU_120 (mmol/L)	0.222	<0.001
HbA1c (%)	0.13	0.011
Insulin (mU/L)	0.006	0.9
HOMA-IR	0.08	0.12
HDL-C (mmol/L)	−0.061	0.23
LogTGs	0.173	0.001
TGs (mmol/L) *	0.168	0.001
LDL-C (mmol/L)	0.077	0.13
Creatinine (mg/dL)	0.014	0.78
eGFR (mL/min/1.73 m^2^)	−0.037	0.47
ALT (U/L)	0.061	0.23
WC (cm)	0.106	0.041
BMI (kg/m^2^)	0.134	0.009
SBP (mmHg)	0.072	0.17
hsCRP nmol/L)	0.008	0.88
FFAs (µM)	0.099	0.052

* Log transformed before analysis. FPG, fasting plasma glucose; GLU_120, glucose level at two hours during an oral glucose tolerance test; HbA1c, glycated hemoglobin A1c; HOMA-IR, homeostasis model assessment of insulin resistance; HDL-C, high-density lipoprotein cholesterol; TGs, triglycerides; LDL-C, low-density lipoprotein cholesterol; eGFR, estimated glomerular filtration rate; ALT, alanine aminotransferase; WC, waist circumference; BMI, body-mass index; SBP, systolic blood pressure; hsCRP, high-sensitivity C-reactive protein; FFAs, free fatty acids.

**Table 3:** Multivariate linear regression analyses of secretogranin III and clinical variables.

Variable	Model 1		Model 2		Model 3	
	β (95%CI)	*p*	β (95% CI)	*p*	β (95% CI)	*p*
Age (years)	−0.062 (−0.049~0.014)	0.28	−0.099 (−0.061~0.006)	0.11	−0.108 (−0.066~0.005)	0.10
Sex (male vs. female)	−0.014 (−0.868~0.675)	0.81	0.027 (−0.617~0.983)	0.65	0.031 (−0.585~1.005)	0.60
MetS (yes vs. no)	0.190 (0.665~2.644)	0.001	0.164 (0.410~2.461)	0.006		
FFAs (µM)			0.179 (0.052~0.260)	0.003	0.149 (0.025~0.234)	0.015
hsCRP (nmol/L)			−0.037 (−0.077~0.040)	0.53	−0.047 (−0.081~0.034)	0.42
Insulin (mU/L)			−0.020 (−0.135~0.096)	0.74	−0.052 (−0.169~0.064)	0.38
eGFR (mL/min/1.73 m^2^)			−0.095 (−0.036~0.004)	0.12	−0.089 (−0.035~0.005)	0.14
ALT (U/L)			−0.021 (−0.034~0.024)	0.72	−0.035 (−0.037~0.020)	0.55
FPG (mmol/L)					0.216 (0.683~2.490)	0.001
WC (cm)					0.133 (0.114~1.897)	0.027
TGs (mmol/L)					0.036 (−0.740~1.343)	0.57
HDL-C (mmol/L)					0.061 (−0.425~1.389)	0.30
BP (mmHg)					−0.006 (−0.858~0.777)	0.92

WC, waist circumference; TGs, triglycerides; HDL-C, high-density lipoprotein cholesterol; BP, blood pressure; hsCRP, high-sensitivity C-reactive protein; eGFR, estimated glomerular filtration rate; ALT, alanine aminotransferase; FPG, fasting plasma glucose; FFAs, free fatty acids.
